# Supplementary material for: DMRforPairs: identifying Differentially Methylated Regions between unique samples using array based methylation profiles
Source: BMC Bioinformatics. 2014 May 15;15:141. doi: 10.1186/1471-2105-15-141 (PMC4046028; doi:10.1186/1471-2105-15-141)
Supplement: Additional file 2 — DMRforPairs output for the comparison of A431-MCF7 and NA17018-NA17105. Please start from the HTML files in each folder. Available via the BMC Bioinformatics website. [file 1471-2105-15-141-S2.zip › 1394847754114233_MOESM2_ESM/A431_MCF7/figures/35756.pdf]

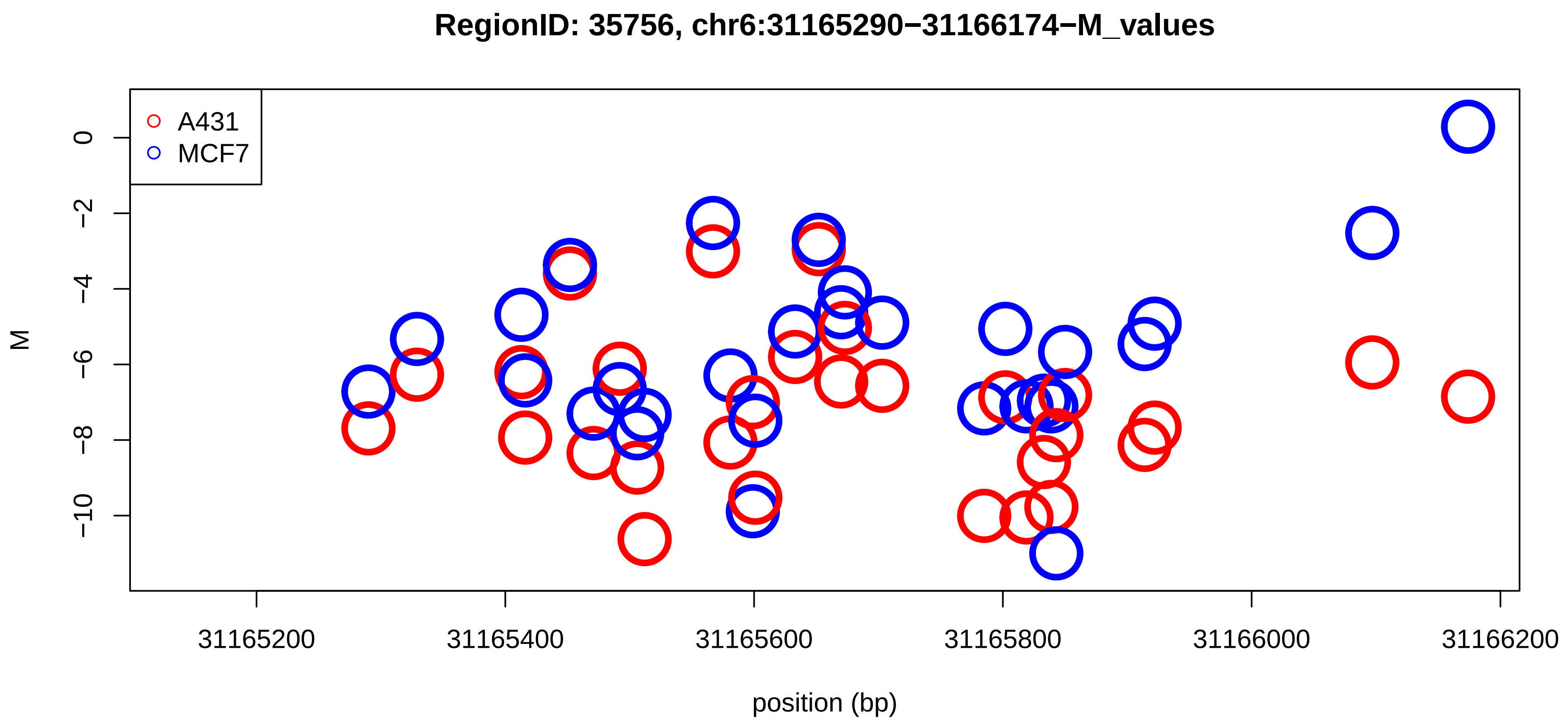
RegionID: 35756, chr6:31165290−31166174−M_values
A431
MCF7
M
0
−2
−4
−6
−8
−10
31165200
31165400
31165600
31165800
31166000
31166200
position (bp)

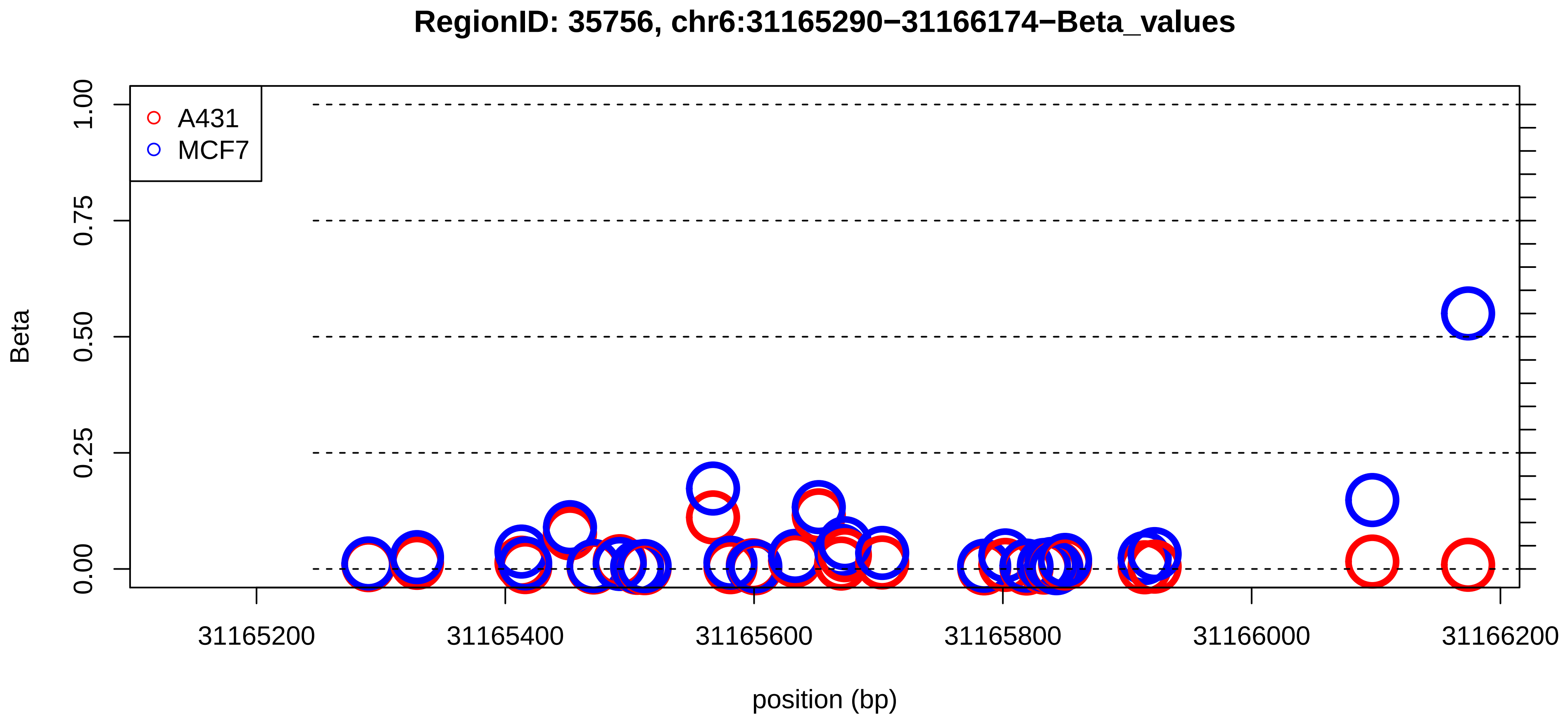
RegionID: 35756, chr6:31165290−31166174−Beta_values
A431
MCF7
Beta
0.00
0.25
0.50
0.75
1.00
31165200
31165400
31165600
31165800
31166000
31166200
position (bp)

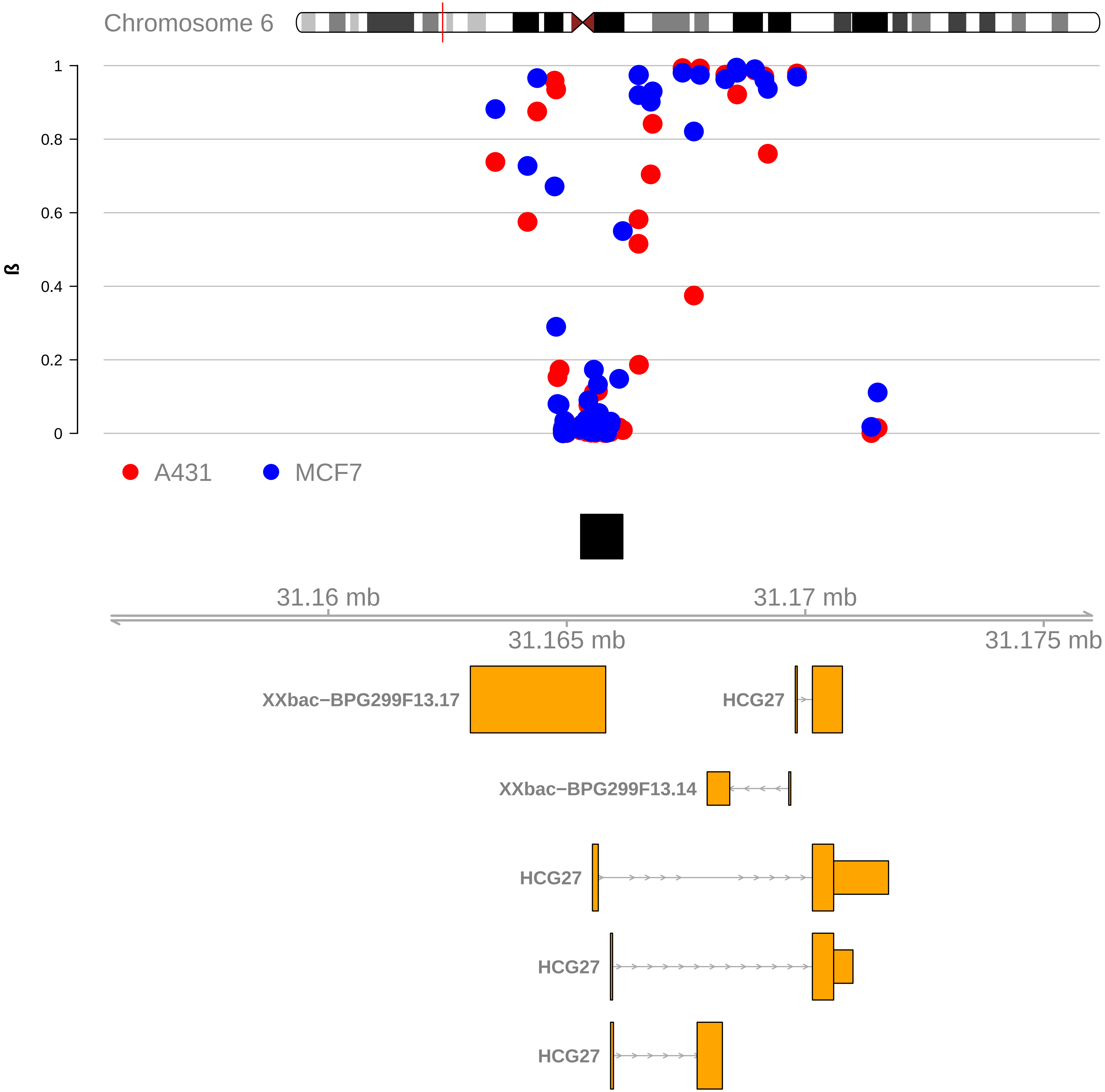
Chromosome 6
ß
1
0.8
0.6
0.4
0.2
0
A431
MCF7
31.16 mb
31.17 mb
31.165 mb
31.175 mb
XXbac−BPG299F13.17
HCG27
XXbac−BPG299F13.14
HCG27
HCG27
HCG27
